# Underestimating isolated bilateral hygroma as non-accidental head injury with dramatic consequences: a case presentation

**DOI:** 10.1007/s00381-022-05720-3

**Published:** 2022-11-03

**Authors:** Gesa Cohrs, Sibylle Maria Winter, Wiebke Siska, Ulrich-Wilhelm Thomale

**Affiliations:** 1 Pediatric Neurosurgery, Campus Virchow Klinikum, Charité-Universitätsmedizin Berlin, Berlin, Germany; 2 Department of Child and Adolescent Psychiatry, Psychosomatics and Psychotherapy, Campus Virchow Klinikum, Charité-Universitätsmedizin Berlin, Berlin, Germany; 3 Child Protection Team, Campus Virchow Klinikum, Charité-Universitätsmedizin Berlin, Berlin, Germany

**Keywords:** Non-accidental traumatic brain injury, Shaken baby syndrome, Abusive head injury, Bilateral hygroma, Subduroperitoneal shunt, Hydrocephalus

## Abstract

**Objective:**

Abusive head injury (AHI) in infancy is associated with significantly worse outcomes compared to accidental traumatic brain injury. The decision-making of the diagnosis of AHI is challenging especially if the clinical signs are not presenting as a multifactorial pattern.

**Method:**

We present a case of isolated bilateral hygroma in which this differential diagnosis of AHI was evaluated but primarily not seen as such leading subsequently to extensive secondary AHI with fatal brain injury.

**Results:**

The case of an 8-week-old infant with apparently isolated bilateral hygroma without any external signs of abuse and no retinal hemorrhages was interpreted in causative correlation to the perinatal complex course of delivery. At a second readmission of the case, severe brain injury with bilateral cortical hypoxia, subarachnoid and subdural hemorrhages, and skull and extremity fractures led to severe disability of the affected infant.

**Conclusion:**

Any early suspicion of AHI with at least one factor possibly being associated with abusive trauma should be discussed in multidisciplinary team conferences to find the best strategy to protect the child. Beside clinical factors, social factors within the family household may additionally be evaluated to determine stress-related risk for traumatic child abuse. In general, prevention programs will be essential in future perspective.

## Introduction

Non-accidental traumatic brain injury also known as abusive head injury (AHI) in infants and young children presents as a complex combination of injuries requiring a multidisciplinary approach. Relevant neurosurgical involvement is required since these children may present initially with bilateral hygroma and increased intracranial pressure, which often require emergency surgical procedures. Recently, substantial progress in understanding any kind of AHI has been made in terms of epidemiology, differential diagnosis, and clinical manifestations, varying in age and trauma mechanism with differing presentation of injury. Nevertheless, prevention programs have not led to substantial reduction in AHI, yet [1–3]. It remains a common problem with an incidence estimated at 15–30/100000 children < 1 year and an overall mortality rate of 15–38% and long-term neurologic deficits in up to 80% of surviving victims, thus associated with significantly worse outcomes compared to accidental traumatic brain injury [4–7]. Furthermore, a recent study has shown a marked increase in AHI incidence and severity during the COVID-19 pandemic, stressing the need for clinical awareness and prevention measures [8]. As a survey among members of the International Society for Pediatric Surgery (ISPN) has shown, this condition is associated with unique challenges and intervention opportunities for neurosurgeons [9]. The survey revealed a wide practice of ICP monitoring and also the use of decompressive craniectomy in this patient cohort. A multidisciplinary approach when suspecting AHI is crucial as the diagnosis can be made in some cases with high confidence; in many others, the clinical findings allow for suspicion but not an assumption of a non-accidental cause. To determine the diagnosis of AHI and the pattern and constellation of injuries, the correlation of the presented patient’s history of trauma with injury patterns and possible multiplicities needs to be taken into account. Up to one third of AHI victims have evidence of a preceding injury, which was not recognized as non-accidental, and they may suffer a more severe or even fatal injury [10–12]. One of the most common findings in AHI is bilateral subdural hemorrhagic hygroma, which also have multiple differential diagnosis in infants such as perinatal and accidental trauma; metabolic and genetic disease; hematologic, oncologic, and autoimmune disease; congenital malformations; and others [13]. Thus, it remains a huge challenge to find the right threshold as well as the right ways of communication if suspicion of AHI needs to be clarified. We present a case of AHI with isolated bilateral hygroma where misinterpretation led to devastating injuries in the further course.

## Case presentation

**Fig. 1:**
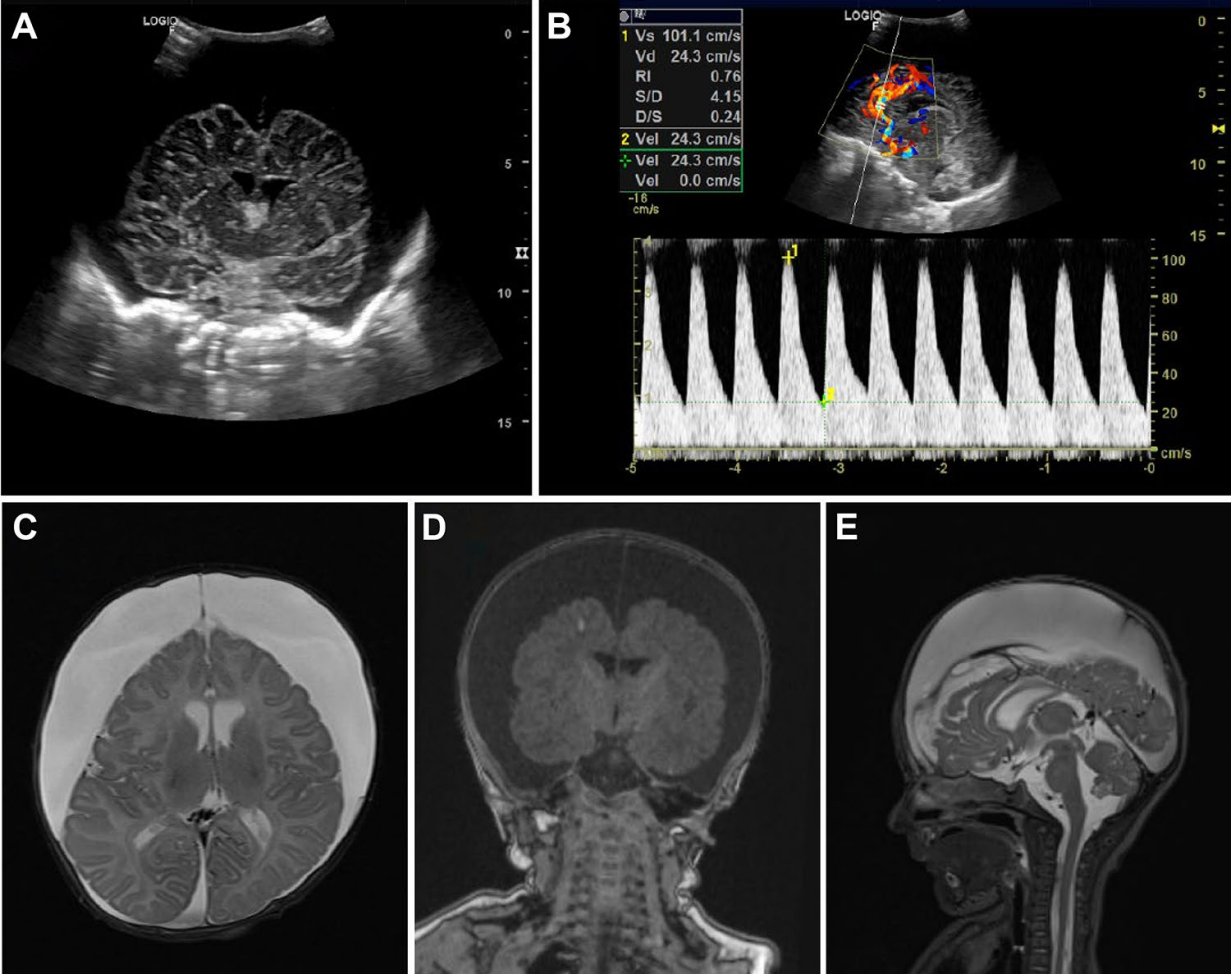
Initial imaging workup after presentation of the patient to the Emergency Department because of macrocephaly, lethargy, and vomiting. **A** Ultrasound scan in the coronal plane, demonstrating extensive bilateral hygroma and **B** the corresponding transcranial Doppler with a resistance index of 0.76. **C** Preoperative MRI with T2-weighted sequence in the axial plane, **D** T1-weighted sequence in the coronal plane, and **E** T2-weighted sagittal plane basically confirming the ultrasound findings

An 8-week-old child was referred from a pediatric department of an external hospital during the summer vacation period because of macrocephaly and bilateral subdural hygroma. The parents had noticed frequent vomiting and lethargy; further, they reported that their child was refusing to be fed. Beforehand, the patient was admitted to an external hospital at the age of 2 weeks and 4 weeks because of impaired feeding and vomiting; the parents also complied all compulsory pediatric follow-up examinations at their pediatrician. The mother had a professional psychosocial background. The child was born at term and delivered at a prolonged course finally accomplished by C-section because of a pathological CTG. The last weeks of pregnancy were complicated by HELLP syndrome. After birth, the parents had noticed a striking cranial deformity. On clinical presentation at admission, the patient was alert, he showed upward gaze palsy, and the head circumference was 43.5 cm (> P99, 3,63z). The child presented with no signs of external injuries suggesting abuse, and the parents denied a history of trauma. The first ultrasound (Fig. 1A, B) revealed extensive bilateral hygroma; the MRI of the head of the following day (Fig. 1C–E) confirmed bilateral hygroma; further, there was minimal intraventricular and subarachnoid hemorrhage as well as slim chronic subdural hematoma (SDH) in the posterior fossa. To clarify possible AHI, an ophthalmologic exam was done, which did not show any retinal hemorrhage. Further, lab workup showed no pathologic results concerning coagulation and blood cell counts; screening for primary coagulopathies and metabolic diseases was negative. The condition birth-related trauma was suspected.

**Fig. 2:**
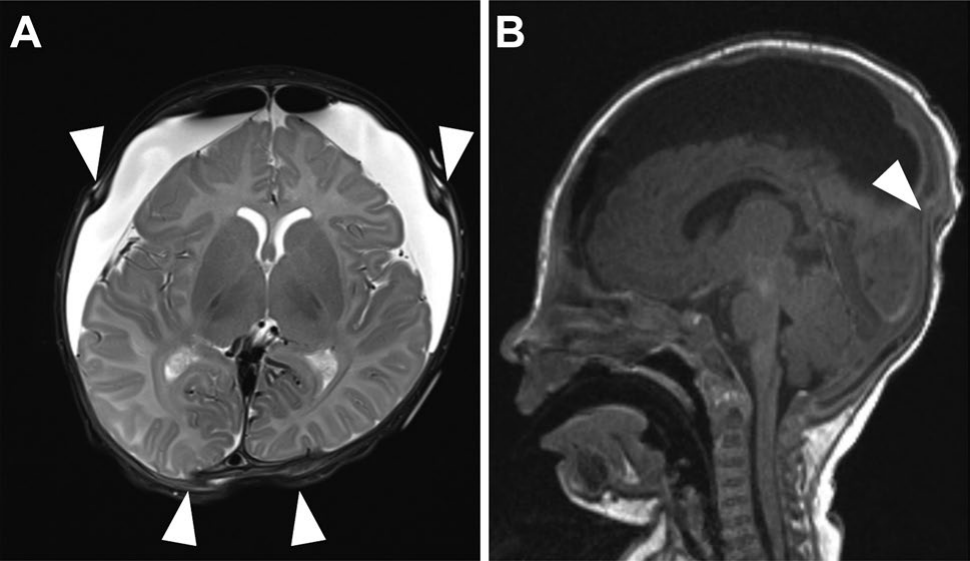
The first postoperative imaging after drainage of the subdural hygroma and insertion of subdural catheter **A** demonstrates the T2-weighted axial and **B** the T1-weighted sagittal MRI depicting the slight displacement of the coronal and lambdoid suture after the release of the subdural fluid (arrowheads) indicating significant pressure relieve

**Fig. 3:**
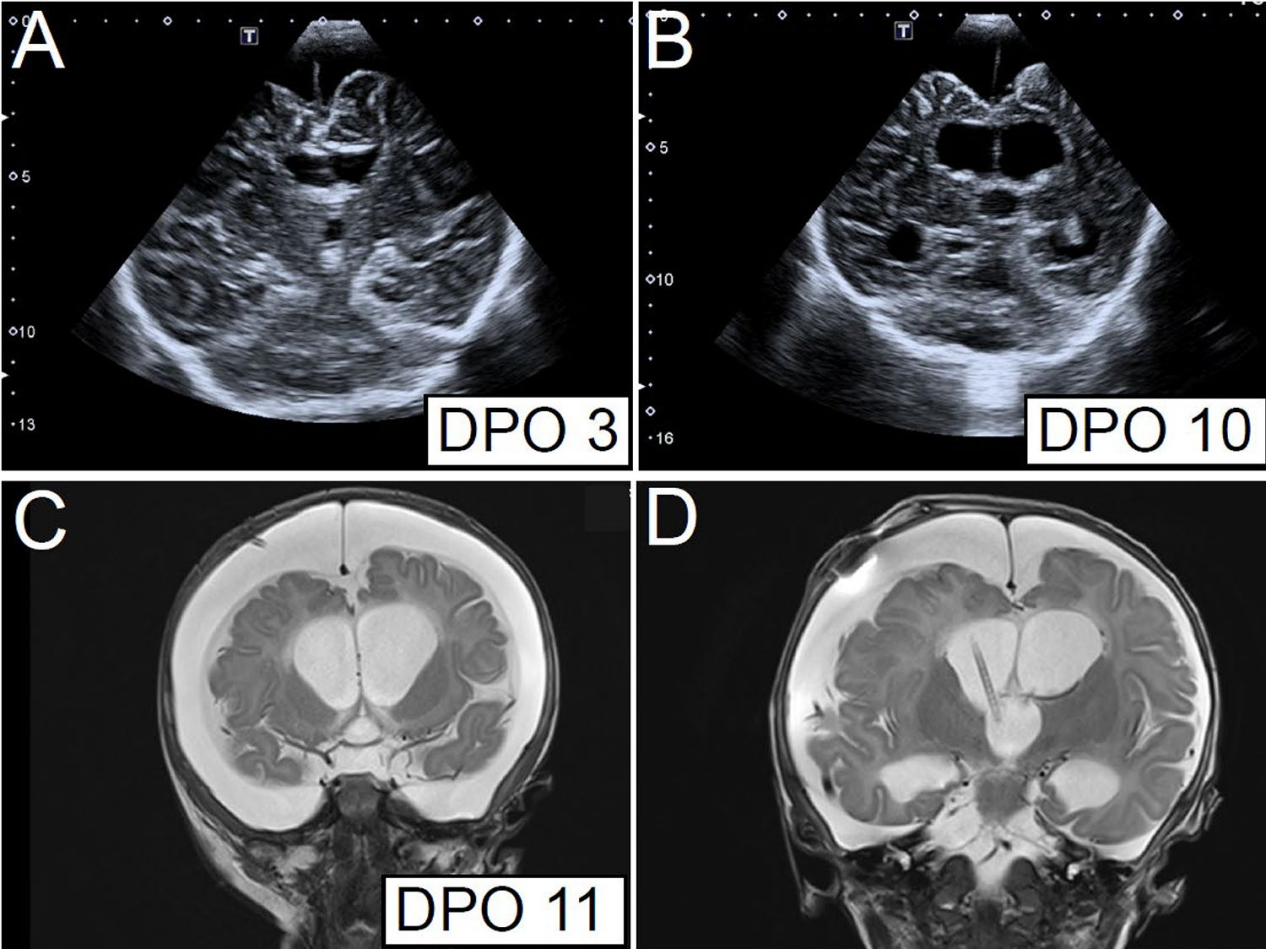
Follow-up imaging indicating dynamic development of hydrocephalus. **A** shows a follow-up coronal ultrasound scan after placement of a subduroperitoneal shunt with smaller but residual subdural hygroma and small ventricles. **B** demonstrates the enlargement of the supratentorial ventricles at the 10th postoperative day; **C** T2-weighted coronal MRI verifies these results one day later. As a consequence, a ventricular catheter was additionally placed and connected to the existing subduroperitoneal shunt system leading to decrease in CSF spaces, respectively, as shown in the coronal plane of the T2-weighted MRI (**D**)

Bilateral burr hole trepanation and insertion of bilateral external drains were performed (postoperative MRI results are shown in Fig. 2); the patient received an endoscopic lavage of the subdural spaces and placement of a subduroperitoneal shunt 5 days after the first operation as the drains kept a high flow rate. He was discharged in an improved neurological status showing a stable head circumference; furthermore, ultrasound in the postoperative follow-up showed improved results (Fig. 3A).

The child was readmitted to our hospital 6 days later because of further increase of 1.5 cm of the head circumference. The ultrasound at admission visualized relevant increase in ventricular size (Fig. 3B), also confirmed by MRI (Fig. 3C), suggesting additional evolvement of hydrocephalus. Therefore, the patient received a shunt revision with additional insertion of a ventricular catheter to the existing subduroperitoneal shunt (Fig. 3D). To enable enhanced drainage of the subdural spaces, the subdural catheter was connected via Y-connector to drain only via the gravitational unit (25 cmH_2_0), while the ventricular catheter was connected to drain via the differential pressure valve (8 cmH_2_0) plus the gravitational unit (25 cmH_2_0). The child showed clear improvement of the neurological status after the shunt revision, MRI and ultrasound showed a decrease of the width of the subdural and ventricular spaces, and the patient was discharged 8 days after the shunt revision.

**Fig. 4:**
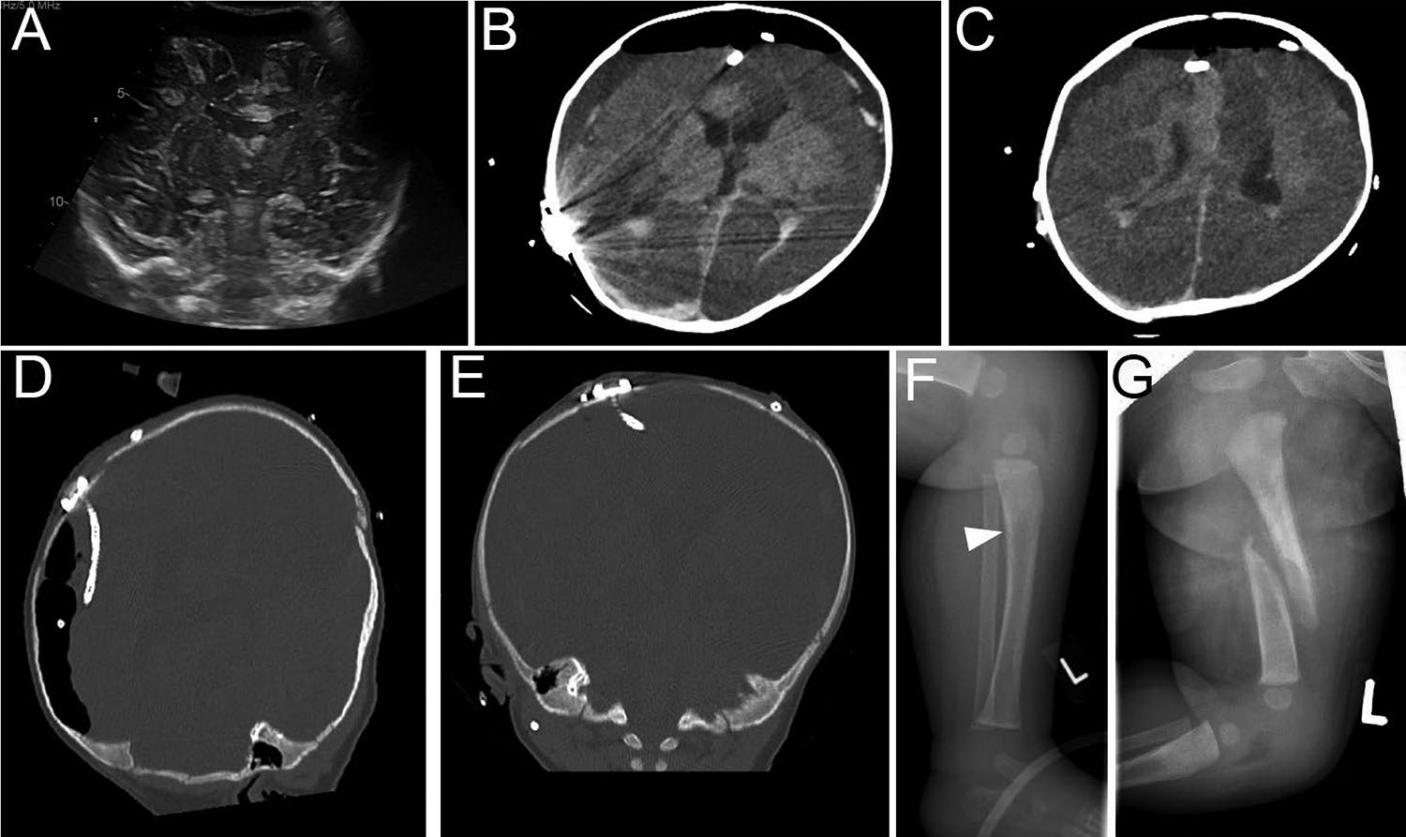
The imaging in the further patients’ course after he was readmitted because of massive clinical deterioration in postictal unconscious state. **A** The cranial ultrasound on admission was rather unspecific, but since clinical signs indicated massively raised intracranial pressure, the reservoir was tapped and high ICP was confirmed. The patient was directly transferred to the OR. The immediate postoperative CT scan was indicating bilateral extensive cortical infarction as hypodensity changes (**B**, **C**). The dislocation of the ventricular catheter on the right side (**D**, **E**) as well as further workup with X-rays identifying fractures of the left tibia in (**F**) and the left femur (**G**) proving severe abusive traumatic injuries

Another 4 days later, the patient was again readmitted to the intensive care unit. The parents repeated reoccurrence of irritability the night before. On the afternoon of admission, epileptic seizures developed with postictal unresponsiveness. The paramedics found the child laying in its bed at GCS of 3 and a dilated pupil on the left side. As the child showed impaired breathing patterns, intubation was performed on site, and the child was transferred to our center. The first ultrasound (Fig. 4A) showed a significantly impaired cerebral diastolic blood flow and the previous known SDH. The burr hole reservoir for the subdural catheter was tapped, showing significant raised intracranial pressure. The relief of the subdural fluid showed a stabilization of the cardiopulmonary situation, and the patient was directly transferred to the OR. Hemorrhagic subdural fluid was drained externally on both sides by placing subdural catheters. The pupil status remained unchanged after surgery. The patient received a CT scan which demonstrated extensive bilateral cortical hypodensities (Fig. 4B, C) and subarachnoid and intraventricular hemorrhage implicating severe brain damage. In addition, a left parietal skull fracture was seen, and dislocation of the ventricular catheter on the right side tilted into the subdural space (Fig. 4D, E). The ophthalmology report of the following day revealed severe bilateral retinal hemorrhage. X-rays of all body parts demonstrated multiple fractures of the proximal tibia and of the femur with dislocation and callus formation (Fig. 4F, G). Subsequently, a MRI was undertaken verifying diffuse ischemic injury and multiple cortical microbleeds. The external drains continuously relieved subdural fluids and could be removed after 13 days. The child protection team of our center initiated further contact to the forensic department and the youth welfare office.

**Fig. 5:**
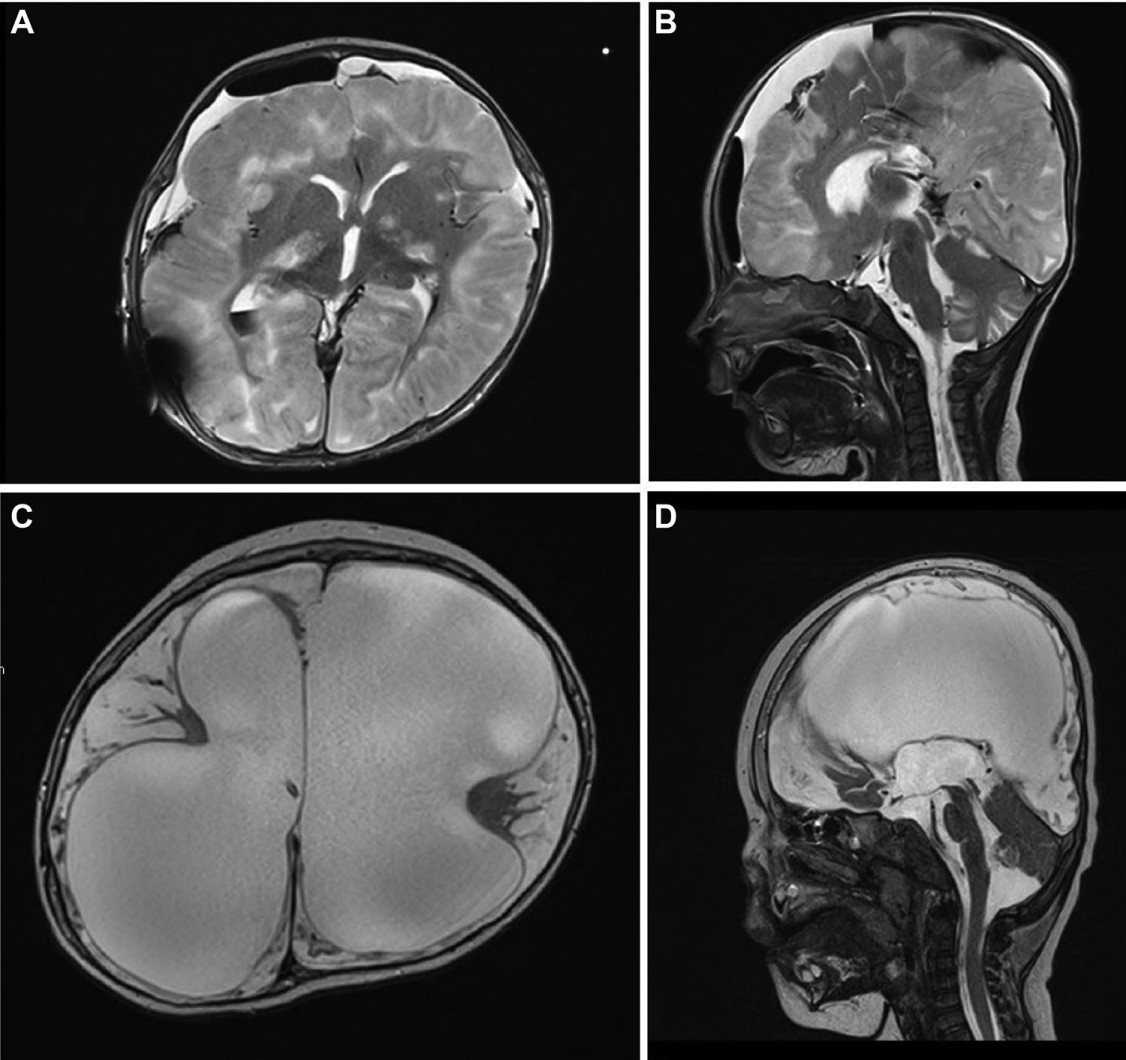
Axial and sagittal T2-weighted MRI sequence showing massive bilateral swelling after hypoxic injury 2 days after the repeated non-accidental trauma (**A**, **B**). T2-weighted MRI at follow-up 1 year after the first presentation, showing hydrocephalus ex vacuo because of extensive encephalomalacia after initial bilateral infarction (**C**, **D**)

In the ongoing course, further epileptic seizures complicated the management. EEG examinations showed severe general dysfunction and epileptic potentials, which were treated with antiepileptic seizure medication. Somatosensory, visually, and auditory-evoked potentials showed severe pathological results. The femur fracture was treated conservatively. Evolving spasticity was treated with baclofen. Extubation of the patient was done 14 days after the admission showing stable breathing patterns. The child was transferred to a rehabilitation center 26 days after admission. The patient received another shunt revision 3 months after discharge from the ICU with replacement of the ventricular catheter and the valve complex due to significant increased head circumference on a palliative basis. The follow-up MRI 1 year after the first admission showed severe brain atrophy and reactively enlarged ventricular system (Fig. 5C, D).

## Discussion

The presented case demonstrates the fatal course of abusive head injury, emphasizing the importance of early intervention and investigation if suspicious facts occur in the history of the patient. It highlights that the threshold when to intervene is crucial for the outcome of affected victims. Physician discomfort or even denial with the diagnosis of AHI and uncertainty can contribute to inaccurate diagnosis. The protection of the child must remain the priority in decision-making. The possible burden to falsely accuse parents must be acknowledged however cannot overrule the child’s safety. Social background or profession of parents has shown to have any prognostic value and may be misleading. The presented case clearly illustrates the vulnerability of young infants, with fatal consequences of repeated abusive head injury usually observed at a median age of 4 months [4]. SDH is the most common pathoanatomical injury encountered in AHI associated with varying degrees of parenchymal brain injury [14, 15]. In the present case, the first lab workup showed no irregularities, and metabolic conditions were ruled out. No signs of trauma to the head or the extremities were found; however, X-rays were not performed since the ophthalmologic examination was normal. Retinal hemorrhages are often a relevant sign of significant abusive trauma, and the severity of the hemorrhages may parallel the severity of neurological trauma. Retinal hemorrhages that are bilateral and severe and include the posterior pole and peripheral hemorrhages are characteristic of AHI [16, 17]. The parents reported about prolonged and complicated delivery with the consequence of a noticeable head shape after birth and some clinical signs developing 2 weeks after birth. As the patient was regularly presented in the outpatient clinic and parents showed compliance concerning any appointments, no further suspicion aroused. However, according to the literature in at least half of AHI cases, no history of trauma is reported, which typically contributes to misdiagnosis. Caregivers may report trauma in some cases, typically a low-height fall in the household which usually does not correlate to the injury patterns [18, 19]. The present case was hospitalized for shunt revision in further course due to hydrocephalus development; in the diagnostic imaging workup, no further trauma-related injuries were detected. Thus, the case seems to be in line with our primary diagnosis of complicated birth-related hygroma development. Finally, it is crucial to ask the question if isolated bilateral hemorrhagic hygroma without signs of external trauma and no signs of retinal hemorrhages must always be handled with suspicion for AHI. In this case, the question must unfortunately be answered positively in retrospect. In the current case, the differential diagnosis of AHI was evaluated, but the formal multidisciplinary team conference was not officially initiated.

The fatality of the case became clear when only days after the shunt revision surgery, the patient was readmitted, in a postictal state, and the diagnostic evaluation showed fatal injuries including extensive hypoxic brain injury, a skull fracture, severe retinal hemorrhages, and multiple extremity fractures (humerus, tibia, femur). Skull fractures in patients with AHI are usually accompanied by intracranial injury and can be identified in up to 25% of AHI victims [18]. There is no specific pattern of skull fracture discriminating an accidental from non-accidental mechanism, but the association with multiple fractures such as rib, spine, and scapular fractures and classic metaphyseal lesions are strong predictors of abuse, when identified in infants with traumatic brain injury [19, 20]. Concerning our center, guidelines are established to detect children at risk at the very first presentation in the emergency room; these guidelines emphasize the importance of an interdisciplinary approach and an early intervention of the Child Protection Team. A transparent multidisciplinary team decision (pediatric psychiatry, social worker, pediatric radiology, pediatric surgeons, ophthalmologists, pediatric neurosurgeons, pediatricians, pediatric intensive care physicians, and forensic medicine experts) must decide about the strategy how to handle the case. Furthermore, the precise documentation of these patients and their injuries is supported by standard documentation forms. The guidelines also comprise information about when families will need social or psychological support at home or if children need to be taken into custody. The initial evaluation of possible stress factors within the family household environment may further help to identify the risk of AHI for the child. After admission of these patients, the child protection team initiates case conferences with the involved departments in order to confirm the diagnosis and will inform administrative authorities. Post hoc discussion of the presented case in a mortality and morbidity conference led to the conclusion that the child protection team needs to be informed at a very low threshold as soon as the differential diagnosis of AHI is raised, and further interdisciplinary conferences concerning these patients should be held in order to develop a solid conclusion on a broad basis from different perspectives. Further efforts must be made to intensify prevention projects. These programs may act right after birth to create awareness for early parenthood that stressful situations of excessive demand may occur, to define coping strategies, and providing information where to receive outside support from early on and at a rather low threshold.

## Conclusion

The current case raises the question of consequent acting if only bilateral hemorrhagic hygroma is diagnosed without any obvious other signs confirming AHI in infants. The fatality of the case with devastating secondary AHI during further course clearly underlines the need for consequent acting if any suspicion for AHI is raised. The consequence is founded in interdisciplinary team evaluations and decision-making in any case of suspicious AHI. Other factors such as social or familial stress in the household environment should be included in this kind of decision-making if only few signs are present to suspect AHI in infants. Further steps need to be taken to establish solid prevention programs. 

## Data Availability

Not applicable.

## Abbreviations

AHI: Abuse head injury
CTG: Cardiotocography
HELLP: Hemolysis, elevated liver enzymes, low platelets
SDH: Subdural hematoma
GCS: Glascow Coma Score
ICP: Intracranial pressure
